# The impact of high and low-intensity exercise in adolescents with movement impairment

**DOI:** 10.1371/journal.pone.0195944

**Published:** 2018-04-26

**Authors:** Francesca Liu, Martyn Morris, Lisa Hicklen, Hooshang Izadi, Helen Dawes

**Affiliations:** 1 Department of Sport and Health Sciences, Oxford Brookes University, Oxford, Oxfordshire, United Kingdom; 2 Department of Biomolecular and Sport Sciences, Coventry University, Coventry, West Midlands, United Kingdom; 3 Department of Kinesiology, University of Texas at Arlington, Arlington, Texas, United States of America; 4 Department of Mechanical Engineering and Mathematical Sciences, Oxford Brookes University, Oxford, Oxfordshire, United Kingdom; 5 Oxford Institute of Nursing & Allied Health Research (OxINMAHR), Oxford Brookes University, Oxford, Oxfordshire, United Kingdom; 6 Department of Clinical Neurology, University of Oxford, Oxford, United Kingdom; 7 Cardiff University, Cardiff, Wales; Rijksuniversiteit Groningen, NETHERLANDS

## Abstract

Five to six percent of young people have movement impairment (MI) associated with reduced exercise tolerance and physical activity levels which persist into adulthood. To better understand the exercise experience in MI, we determined the physiological and perceptual responses during and following a bout of exercise performed at different intensities typically experienced during sport in youth with MI. Thirty-eight adolescents (11–18 years) categorised on the Bruininks-Oseretsky Test of Motor Proficiency-2 Short-Form performed a peak oxygen uptake bike test ($\dot{\text{V}}{\text{O}}_{2\text{peak}}$) test at visit 1 (V1). At visits 2 (V2) and 3 (V3), participants were randomly assigned to both low-intensity (LI) 30min exercise at 50% peak power output (PPO_50%_) and high-intensity (HI) 30s cycling at PPO_100%_, interspersed with 30s rest, for 30min protocol (matched for total work). Heart rate (HR) and rating of perceived exertion (RPE) for legs, breathing and overall was measured before, during and at 1, 3 and 7-min post-exercise (P1, P3, P7). There was a significant difference in $\dot{\text{V}}{\text{O}}_{2\text{peak}}$ between groups (MI:31.5±9.2 vs. NMI:40.0±9.5ml⋅kg^-1^⋅min^-1^, p<0.05). PPO was significantly lower in MI group (MI:157±61 vs. NMI:216±57 W)(p<0.05). HR_avg_ during HI-cycling was reduced in MI (140±18 vs. 157±14bpm, p<0.05), but not LI (133±18 vs. 143±17bpm, p>0.05). Both groups experienced similar RPE for breathing and overall (MI:7.0±3.0 vs. NMI:6.0±2.0, p>0.05) at both intensities, but reported higher legs RPE towards the end (p<0.01). Significant differences were found in HR_recovery_ at P1 post-HI (MI:128±25.9 vs. NMI:154±20.2, p<0.05) but not for legs RPE. Perceived fatigue appears to limit exercise in youth with MI in both high and low-intensity exercise types. Our findings suggest interventions reducing perceived fatigue during exercise may improve exercise tolerance and positively impact on engagement in physical activities.

## Introduction

Motor coordination deficits and inefficient movement patterns are notable contributors to the reduced exercise capacity exhibited in individuals with movement impairment (MI). Nearly 2.6 million people in the UK [1] and an estimated 6% worldwide [2] present with movement difficulties including developmental coordination disorder (DCD) and neurodevelopmental/neurological conditions (i.e., cerebral palsy). Children and adolescents with MI often display insufficient physical activity (PA) levels [3–6] compared to typically developing (TD) peers and correspondingly engage at lower intensities when participating in sports and play [7]. According to the World Health Organization (WHO), individuals between 5–17 years old should accumulate at least 60 min of moderate-to-vigorous physical activity (MVPA) daily and incorporate vigorous-intensity PA three days per week [8], in order to confer positive health effects [9]. Consequently, decreased levels of PA have implications for many aspects of children’s physical and cognitive development [10] and general health and well-being [3]. Adolescents with MI have also demonstrated higher rates of obesity compared to TD, and as a result may lead to an increased risk for developing metabolic syndrome [11, 12]. Of added concern is that such motor impairments and poor coordination contribute to a vicious cycle of reduced enjoyment, tolerance and participation [13, 14], which is known to persist throughout adolescence into adulthood [15]. However, understanding why these young people fail to meet recommended PA levels remains a complex phenomenon influenced by a multitude of factors [16–18].

Major factors associated with reduced PA participation in MI have been reported to relate to exercise-induced symptoms of muscle fatigue, poor physical tolerance and lower energy levels [14, 17]. This is further evidenced in the literature whereby young people with motor coordination difficulties and MI commonly exhibit lower fitness (including aerobic power, muscle strength, endurance, anaerobic power) [19, 20]. One explanation may be that children with MI experience earlier symptoms of fatigue compared to motorically proficient peers [17]. Children and adolescents with higher levels of MI have also demonstrated an inability to exercise hard enough to tax the cardiovascular system despite their ability to push themselves and stress their muscles to work anaerobically during exercise [13]. Moreover, it has been suggested that hypoactivity presented in youth with MI, is associated with lower self-perception and poor self-adequacy [14] and may be a significant determinant in predicting engagement in PA during adolescence [5].

Aerobic moderate PA is a powerful stimulus for improving cognition [21] and is undisputedly evidenced to improve fitness, health and wellbeing [8]. Both lower-intensity PA [22] and moderate-vigorous intensity activity approaches have equally demonstrated favorable health benefits in TD. However, based on the findings by Morris et al. [13], utilising short duration, HI exercise may provide a feasible method for targeting aerobic capacity and improving fitness parameters in MI. There is emerging evidence supporting the notion that HI exercise may serve as an effective strategy to increase muscle power and improve exercise capacity [16], allowing untrained individuals to work harder than would otherwise be possible at a steady-state intensity [16, 23]. Intermittent exercise is hypothesised to be easier for less trained individuals whilst inducing similar or even superior physiological benefits on oxidative capacity and endurance performance [18]. Several studies have already highlighted the positive effects of high-intensity training (HIT) on improving measures of physical fitness and cardiometabolic risk factors [16, 23, 24] in TD children. In comparison to moderate intensity levels the potential advantages of HIT are the purported time-efficiency of the exercise modality and the enjoyment associated with this form of training [16, 25]. Despite the renewed popularity and interest in HI exercise, there is limited information regarding the potential injury risks and adverse responses in youth performing higher intensities, specifically in children with MI.

According to the literature, MI may be associated with inefficient movement patterns [4, 14, 26] contributing to poorer physical tolerance and fatigue, which can be defined as an acute impairment of exercise performance that includes both an increase in perceived effort required to produce a power output and the eventual inability to maintain power output [27]. Fatigue has been highlighted as a major factor affecting exercise performance in youth with MI [14, 17]. To date, the limiting factors surrounding exercise capacity in children with MI have been suggested to be peripherally derived (e.g., local muscle fatigue) [13] and/or due to inefficiencies in the oxygen transport system (e.g., inadequate cardiac output) [13, 26, 28]. Perceptual factors are also known to limit exercise [29] as confirmed by pain and discomfort ratings increasing with RPE when performing both progressive and interval exercise [30] in children and adults. During activity, RPE represents an integration of information concerning previous experience, whereby the self-reported changes in effort reflect the physiological and psychological processes that under certain conditions induce fatigue [31]. To this regard, the physiological and perceptual responses during either continuous or intermittent and more specifically with HI or LI activities have not been described in young people with MI. A better understanding of the exercise responses in these individuals is an important first step to help us identify how to support these children to perform at higher intensities and engage in more physical activity.

## Aims

The purpose of this study is to describe exercise responses to maximal exercise and explore the extent of the physiological and perceptual responses during and following an acute cycling bout of work-matched HI and LI exercise in children and adolescents with MI compared to no-movement impairment (NMI).

## Materials and methods

### Procedure

This was a randomised crossover study, utilising two acute exercise exposure conditions and approved by the University Research Ethics Committee (UREC Registration No. 130773). Participants were recruited from local schools and a local Clinical Exercise and Rehabilitation Unit (CLEAR) in Oxfordshire, UK. Individuals attending the CLEAR unit include children with MI, DCD and neurological disabilities who participate in weekly gym sessions. Families indicating that they were interested in taking part were sent separate child and parent information sheets and gave their written consent prior to participating in the study. Participants attended the Movement Science Laboratory for testing on three separate occasions, with approximately seven days between each visit. Participants were asked to refrain from eating, performing exercise or drinking caffeine in the 2 h period before attending the sessions. All participants were fully familiarised with the testing protocol prior to data collection.

### Participants

Forty-three adolescents aged 11–18 years with no known neurological condition were recruited. For the inclusion criteria, participants were required to be able to walk with or without support for at least five meters and be able to safely take part and follow a two-step instruction during testing procedures. Participants with any known contraindications to exercise participation (i.e., muscular degenerative conditions, congenital heart disease, uncontrolled exercise-induced asthma, chronic obstructive pulmonary disorder, uncontrolled epilepsy/on medication for ≤12 weeks) were ineligible to take part. Furthermore, participants presenting with attentional, learning and/or mental health conditions as indicated by their parents and on the information sheets were excluded from this study due to potential confounders when examining perceptual responses.

### Measures

#### Baseline measures

Baseline measures including level of movement impairment (BOT-2 SF) and $\dot{\text{V}}{\text{O}}_{2\text{peak}}$ were used to classify individuals in the MI and NMI groups and to ascertain PPO for assignment of intensity levels (HI and LI). Assessment of movement economy or muscular efficiency ($\dot{\text{V}}{\text{O}}_{\text{2}}$/W), cardiac efficiency (O_2_ pulse), HR_peak_ and RER were also measured from the $\dot{\text{V}}{\text{O}}_{2\text{peak}}$ test to establish baseline fitness parameters.

#### Bruininks-Oseretsky Test of Motor Proficiency 2 Short Form (BOT-2 SF)

During the first visit, the BOT-2 SF, a standardised test of motor proficiency was used to categorise level of movement impairment [32]. Four motor area composites were included in the BOT-2 SF encompassing; fine motor control, manual coordination, body coordination and strength and agility. Thirteen items were individually administered as described in the test manual [32]. Raw scores for each task were converted to a point score under each subtest and summed across to obtain a total standard score. The total standard scores were compared to normative scores and age equivalents to determine the individual’s percentile rank and to describe overall motor skill proficiency level. Based on the BOT-2 SF manual [32], individuals scoring below the 17^th^ percentile cut-off were considered to have lower motor skill proficiency [32] and categorised as MI and individuals scoring >17^th^ percentile were indicated as having no-movement impairment (NMI).

### Exercise testing

Height (m) (Holtain stadiometer), weight (kg) (Seca scales), body mass index (BMI) (kg/m^2^) and sexual maturation [33] were recorded prior to the exercise test at V1. For the purposes of testing individuals with a wide range of movement abilities, measurement of peak oxygen uptake ($\dot{\text{V}}{\text{O}}_{2\text{peak}}$) was performed with an incremental step test on a cycle ergometer (Lode Excalibur Sport, Groningen, The Netherlands). The protocol consisted of 1 min stages after an initial 2 min of unloaded cycling. Workload was progressed by 15–20 Watt (W) from unloaded cycling each minute based on the height of the participant [34]. The test was terminated when the participant reached volitional exhaustion or was unable to maintain a cadence of 60 revolutions per minute (rpm) despite verbal encouragement. Oxygen uptake ($\dot{\text{V}}{\text{O}}_{\text{2}}$), carbon dioxide produced ($\dot{\text{V}}{\text{CO}}_{\text{2}}$) and volume of expired air per minute (${\dot{\text{V}}}_{\text{E}}$) were measured breath-by-breath using an online gas analyser (Cortex Metalyser 3B, Cortex, Leipzig, Germany). Before each testing session, the gas analysers were calibrated according to manufacturer guidelines. The gas sample line was calibrated using gases of a known concentration and flow volume was calibrated using a 3 L syringe (Hans Rudolph). All participants wore a fitted face-mask covering the nose and mouth connected to a low resistance volume transducer (Triple V, Hoechberg, Germany). Additionally, heart rate (HR) was recorded continuously throughout the testing using short-range telemetry (Polar S810, Finland). Oxygen uptake ($\dot{\text{V}}{\text{O}}_{\text{2}}$) was recorded as the highest 30 s average of each stage, while $\dot{\text{V}}{\text{O}}_{2\text{peak}}$ was recorded as the highest 30 s average before the termination of the test. The criteria for obtaining a $\dot{\text{V}}{\text{O}}_{2\text{peak}}$ was considered when two of the three following criteria was achieved: 1) HR >180 beats/min [35], 2) Respiratory exchange ratio (RER) >-1.06 [36], and/or 3) subjective signs of exhaustion [37]. The PPO was determined as the highest workload (W) attained at $\dot{\text{V}}{\text{O}}_{2\text{peak}}$ for the completed stage.

The RER was calculated from the ratio of $\dot{\text{V}}{\text{O}}_{\text{2}}$ to $\dot{\text{V}}{\text{CO}}_{\text{2}}$ at each workload level throughout the exercise test. For the measurement of muscular efficiency or the relationship between the amount of oxygen utilised for a given work rate, the linear slope of the relationship between $\dot{\text{V}}{\text{O}}_{\text{2}}$ and W ($\dot{\text{V}}{\text{O}}_{\text{2}}$/W) was derived. Oxygen pulse (O_2_), a non-invasive indicator of cardiac efficiency, was also calculated by dividing $\dot{\text{V}}{\text{O}}_{2\text{peak}}$ by HR_peak_ ($\dot{\text{V}}{\text{O}}_{2\text{peak}}$/HR_peak_) and expressed as mL/beat. Rating of perceived exertion (RPE) was measured at the end of each stage using the Cart and Load Scale (CALER), which has previously been used to assess children’s perception of effort during exercise [38].

### Exercise interventions

**Fig 1:**
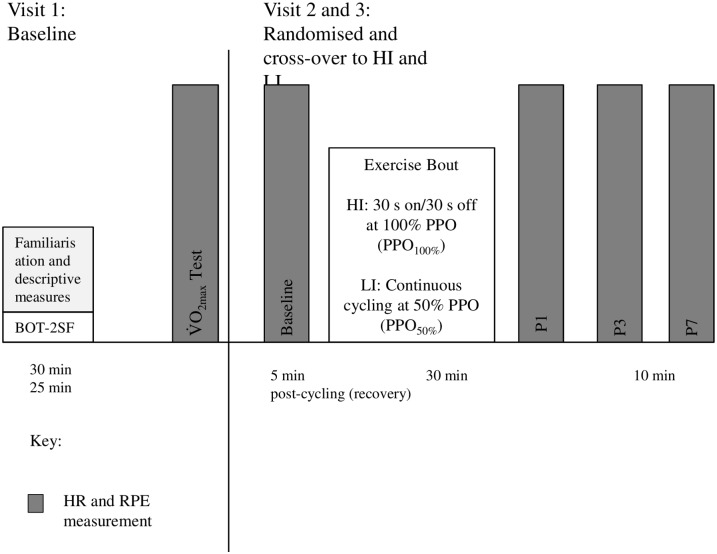
Diagram of the three-visit study protocol. BOT-2 SF, Bruininks-Oseretsky Test of Motor Proficiency Short Form 2; HI, High-intensity; LI, Low-intensity; PPO, peak power output; P1, post-1 min; P3, post-3 min; P7, post-7 min $\dot{\text{V}}{\text{O}}_{2\text{max}}$, Peak oxygen uptake.

Participants were asked to complete two experimental conditions in a randomised crossover design for the exercise intervention including: a HI-cycling bout and a LI-bout of cycling (Fig 1).

The exercise was performed on a cycle ergometer (Lode Excalibur Sport, Groningen, The Netherlands) with the bike seat height adjusted to the participants’ comfort and recorded for subsequent sessions. Participants performed the exercise at the same time of day for each session under standard temperature conditions in the laboratory (~20–22 °C). For the HI session, participants were asked to perform a 30 min bout of cycling consisting of pedaling for 30 s-on, then no pedaling for 30 s-off at 100% PPO (PPO_100%_) as determined from PPO during the maximal incremental bike test. In contrast, for the LI session participants were asked to cycle continuously for 30 min at PPO_50%_. Throughout the session, both HR (Polar S810, Finland) and RPE (CALER scale) was monitored and recorded at 5 min intervals. Participants were asked to rate their RPE for legs, breathing and overall using the CALER scale every 5 min throughout the 30 min session. Following each session, participants were informally asked to indicate enjoyment level.

#### Cardiovascular and perceptual outcome measures

Outcomes measures of interest include cardiovascular (HR) and perceptual (RPE) responses following both HI- and LI-cycling bouts. Additional measures of interest include heart rate average (HR_avg_) and HR recovery (HR_recovery_) at post-1, 3 and 7 min exercise (P1, P3, P7). When considering relative HR, this was represented as percentage change (%Δ) from HR_peak_, which was expressed as percentage of the maximal heart rate (HR_max_) throughout the study (i.e., %HR_max_). The %HR_max_ has been used extensively as prescription means of exercise intensity and exemplifies a close relationship with oxygen uptake [39]. Comparisons between groups (MI vs. NMI), between conditions (HI vs. LI) and across time points (average during 30 min bout and post-exercise P1, P3, P7) were made to assess the impact of exercise intensities on outcome measures as well as the interactions between group and time.

### Sample size

Based on statistical power calculations using G*Power 3.1.9.2 (Heinrich Heine University Düsseldorf, Germany), a sample size of 45 participants total is required for a power of 0.95, an alpha (α) of 0.05 and an effect size (Cohen’s d) of 0.50 to detect differences between the group means.

### Randomisation

Participants were randomised using an excel program which generated a random allocation to either the HI- or LI-bout first. The researchers informed the participant which exercise intensity they would perform for the second session (V2) and then cross-over to the other intensity bout for the third and final session (V3).

### Data analysis

All data are presented as mean ± SD. Statistical analyses were performed in SPSS for Windows v21 (SPSS Inc., Chicago, IL, USA). Normality of the data was checked by Shapiro Wilk tests. Homogeneity of variances was confirmed by Mauchley’s test of sphericity and a Greenhouse-Geisser correction was applied to the degrees of freedom if the sphericity assumption was violated. Baseline exercise measurements ($\dot{\text{V}}{\text{O}}_{2\text{peak}}$, HR_max_, RER, RPE, PPO/W) were analysed using student’s t-test. A Pearson correlation coefficient (r) was used to examine the linear relationship between BOT-2 SF scores and baseline $\dot{\text{V}}{\text{O}}_{2\text{peak}}$. Within-session exercise measurements (HR, RPE) were analysed using a linear mixed model (LMM) [40] for repeated measures over time by group to analyze the impact of the different exercise intensities (HI vs. LI) on outcome measures at baseline, during and post-exercise with fixed effects of group (MI vs. NMI), time (HI and LI session) and the interactions between group and time. This method prevented listwise deletion due to missing data [41]. A scatter plot of the predicted values on the x-axis and the residuals on the y-axis were plotted to visually check for linearity whereby no obvious pattern should be displayed and outliers were identified. Furthermore, a Shapiro-Wilk test was performed to determine normality of residuals. Since only two repeats were assessed, an unstructured covariance structure was utilised.

## Results

**Fig 2:**
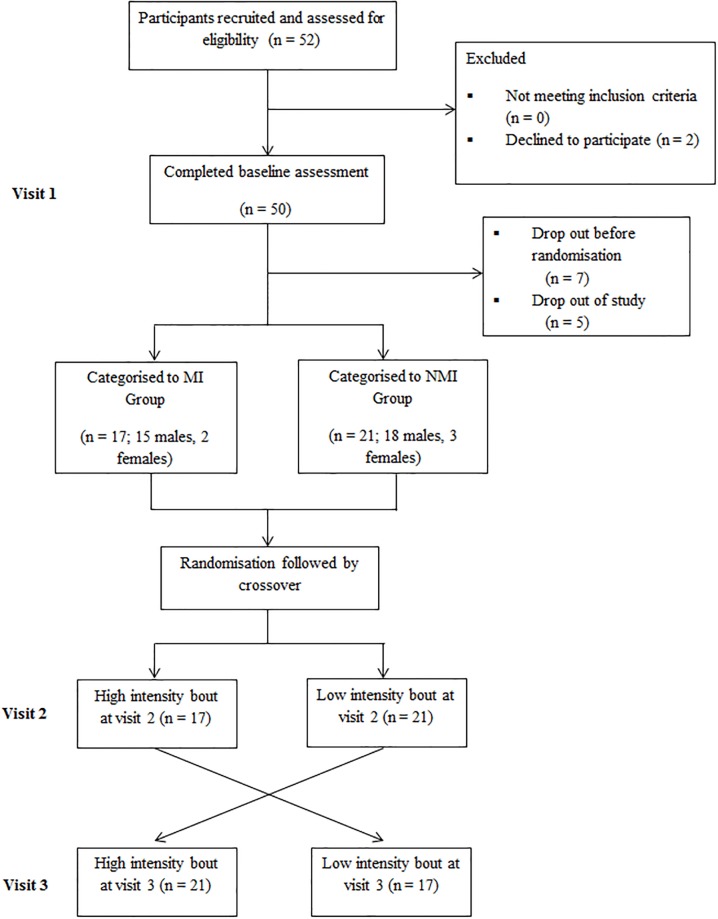
Flow diagram of participant recruitment and adherence throughout study. MI, movement impairment; NMI, no-movement impairment.

A total of 43 adolescents volunteered to participate in this study (Fig 2), with results presented for 38 (11–18 years) as five of the participants were unable to complete all visits required for the study.

The participants were classified into two levels of movement impairment according to the BOT-2SF: those with MI (n = 17; 15 males, 2 females) and those who were normally coordinated, with NMI (n = 21; 18 males, 3 females). Baseline characteristics of participants are presented in Table 1.

**Table 1:** Baseline participant characteristics (mean ± SD).

N = 38	MI (n = 17)	NMI (n = 21)	*P-value
Age (years)	14.5 ± 2.0	15.5 ± 2.0	-
Height (m)	1.70 ± 8.6	1.74 ± 10.6	-
Weight (kg)	63.3 ± 15.6	66.4 ± 16.3	-
BMI (kg/m^2^)	22.0 ± 0.0	22.0 ± 0.0	-
Tanner	5.0 ± 0.0	5.0 ± 0.0	-
BOT-2SF Raw Score	61.0 ± 6.0*	71.0 ± 18.0	p < 0.05
BOT-2SF Standard Score	36.0 ± 2.0*	44.0 ± 12.0	p < 0.05

BMI, body mass index; BOT-2 SF, Bruininks-Oseretsky Test of Motor Proficiency Short-Form 2; kg, kilogram; m, metre; Tanner, Tanner Scale of Sexual Maturity.

*p ≤ 0.05 vs. NMI at baseline.

### Baseline measures

Seventeen of the participants considered to be MI scored below the BOT-2 SF 17^th^ percentile (below average) and 21 were classified as having NMI (BOT-2 SF >17^th^ percentile). Table 1 illustrates the participant characteristics from the motor proficiency assessment. There was a significant difference in the BOT-2 SF standard score between MI (36.0±2.0) and NMI (44.0±12.0) [95% CI: -14.18, -1.61; p<0.001]. Moreover, there was a significant relationship between BOT-2 SF score and $\dot{\text{V}}{\text{O}}_{2\text{peak}}$ (r = 0.62, p<0.05) in both groups and a significant difference in $\dot{\text{V}}{\text{O}}_{2\text{peak}}$ between groups (MI: 31.5±9.2 vs. NMI: 40.0±9.5 ml·kg^-1^·min^-1^) [t(36) = -2.28, p<0.01, 95% CI: -1.08, -0.29]. The PPO (W) was significantly lower in the MI group (MI: 157.0±61.0 vs. NMI: 216.0±57.0 W) [t(33) = -3.05, p<0.01, 95% CI: -101.1, -20.12] and for the LI workload (MI: 85.0±38.0 and NMI: 121.0±29.0 W) [t(31) = -2.38, p<0.05, 95% CI: -51.4, -3.99]. With regard to assessment of movement economy, the MI group demonstrated greater inefficiencies during the maximal exercise test ($\dot{\text{V}}{\text{O}}_{\text{2}}$/PO) (MI: 13.3±3.0; NMI: 11.2±2.0 mL/W) [t(21) = 2.12, p<0.05, 95% CI: -2.85, 2.18] (Table 2). However, HR_max_ was similar between groups (MI: 170.0±25.0 and NMI: 180.0±17.0 bpm) [t(35) = -1.31, p>0.05, 95% CI: -24.7, 5.48] and there were no significant differences in O_2_ pulse (MI: 19.0±0.03; NMI: 20.0±0.05 mL/beat) [t(36) = -0.34, p>0.05, 95% CI: -1.79, 1.30] (Table 2). Correspondingly, there was no difference in the perception of effort throughout the exercise test and at exercise termination. All participants reported an RPE rating of 9 or 10 at the end of the test despite the MI group demonstrating significantly lower PPO at the end of the incremental bike test as shown below in Table 2. Overall, each participant exhibited a RER_max_ greater than 1.06 at the end of the test, however, there was a significant difference between the groups for the RER_max_ value (MI: 1.20±0.20 and NMI: 1.34±0.10) [t(21) = -2.61, p = 0.008, 95% CI: -2.06, 0.88].

**Table 2:** Baseline exercise intensity descriptors (mean ± SD).

N = 38	MI (n = 17)	NMI (n = 21)	*P-value
$\dot{\text{V}}{\text{O}}_{2\text{peak}}$ (L/min)	1.90 ± 0.49*	2.39 ± 0.78	p < 0.05
$\dot{\text{V}}{\text{O}}_{2\text{peak}}$ (mL/kg·min)	31.54 ± 9.2*	36.0 ± 11.0	p < 0.05
HR_max_ (bpm)	170.0 ± 25.0	180.0 ± 17.0	-
PPO (W)	157.0 ± 60.5*	216.0 ± 57.0	p < 0.05
$\dot{\text{V}}{\text{O}}_{\text{2}}$/workload (mL/W)	13.3 ± 4.0*	11.2 ± 2.0	p < 0.05
O_2_ pulse (mL/beat)	19.0 ± 0.03	20.0 ± 0.05	-
RPE overall	9.00 ± 1.0	8.00 ± 1.00	-
RER	1.20 ± 0.20*	1.34 ± 0.10	p < 0.05
HI workload/PO (W)	145.0 ± 65.0*	216.0 ± 69.0	p < 0.05
HI HR_avg_ (bpm)	139.0 ± 18.0*	156.0 ± 13.0	p < 0.05
%Δ HI_baseline_	82.0 ± 18.0	87.0 ± 14.0	-
LI workload/PO (W)	80.0 ± 30.0*	108.0 ± 36.0	p < 0.05
LI HR_avg_ (bpm)	134.0 ± 18.0	143.0 ± 16.0	-
%Δ LI_baseline_	78.0 ± 18.0	79.0± 16.0	-

Avg, average; bpm, beats per minute; HR_max_, heart rate maximum; HI, high intensity; LI, low intensity; L, Litre; mL, milliltre; movement impairment; NMI, no-movement impairment; O_2_ pulse, Oxygen pulse; PPO, peak power output; RER, respiratory exchange ratio; RPE, rating of perceived exertion; VO_2peak_, peak oxygen uptake; $\dot{\text{V}}{\text{O}}_{\text{2}}$/PO, muscular efficiency; W, Watt; W_peak_ watt max; %Δ HI_baseline_, percentage change from baseline at HI (100%); %Δ LI_baseline_, percentage change from baseline LI (100%).

*p ≤ 0.05 vs. NMI at same time point.

### Cardiovascular and perceptual responses

**Fig 3:**
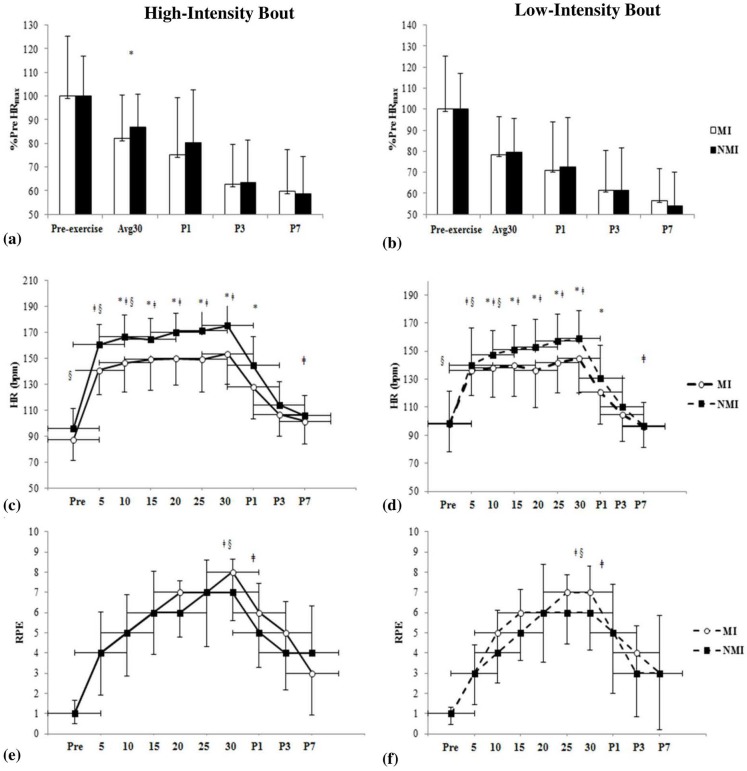
Cardiovascular and perceptual responses for high- and low-intensity bouts. Percent change from baseline heart rate maximum (HR_max_) during cycling bout and post-1, 3 and 7 min (P1, P3, P7) presented for high-intensity visit (HI) (a) and low-intensity visit (LI) (b). MI group (Hollow bars) and NMI group (filled bars). Figs (c) and (e) illustrate the change in HR and ratings of perceived exertion (RPE) pre, during and post-HI-cycling (solid line). Measures were recorded every 5 min throughout the cycling and following in recovery at P1, P3and P7. Figs (d) and (f) represent HR and RPE during the LI-cycling bout (dotted line). Vertical and horizontal error bars represent standard deviation (SD). *p≤0.05 vs. NMI (group); ǂp≤0.05 for Intensity; §p ≤0.05 vs. NMI at same time point (Group x Intensity).

Mean workload during the LI-cycling bout was 85.0±38.0 W in the MI group and 121.0±29.0 W in the NMI group (p<0.05). There was a significant difference between groups [F(1,36.1) = 7.1, p = 0.012] and an effect of intensity for HR_avg_ as demonstrated by the LMM [F(1,33.3) = 37.0, p<0.001]. Overall, HR_avg_ during HI-cycling, which took into account an average of each cycle (i.e., 30s on and 30s off) throughout the 30 min duration, was lower in MI compared to NMI (140.0±18.0 and 157.0±14.0 bpm, p<0.05) [t(34) = -3.28, p = 0.002], but not during LI-cycling (133.0±18.0 and 143.0±17.0 bpm, p>0.05) [t(33) = -1.64, p>0.05]. This denotes that there is a significant difference with regard to how the level of intensity affects the two groups with the MI group experiencing less HR_avg_ variability irrespective of intensity. When considering relative HR represented as percentage change (%Δ) from HR_max_ (% HR_max_), significant differences were only demonstrated during an average of the 30 min cycling bout for HI (MI: 82.0±9.5 vs. NMI: 87.0±7.1%) [t(36) = -3.21, p = 0.003] (Fig 3(a) and 3(b)). In contrast, the MI group did not demonstrate a great deal of change from HI during the LI bout (MI: 78.0±19.3 vs. NMI: 79.0±23.4%) [t(36) = -0.27, p>0.05]. To further validate the findings, a paired samples t-test showed no significant difference in HR_avg_, however, relative change was significantly different in NMI [t(20) = 2.73, p = 0.013].

In the recovery phase, HR at post-1 min (P1) was significantly different for group following HI (MI: 128.0±24.2 vs. NMI: 145.0±22.3 bpm) and LI (MI: 121.0±23.0 vs. NMI: 131.0±23.4 bpm) [F(1,35.2) = 4.91, p<0.05]. No significant differences in HR for group were observed at P3 for either intensity, however, significant differences for intensity were exhibited at P7 following HI (MI: 102.0±17.2 vs. NMI: 106.0±15.4 bpm) and LI (MI: 96.0±15.2 vs. 97.0±16.2 bpm) [F(1,26.4) = 9.80, p = 0.004] and illustrated in Fig 3(c) (HI bout) and Fig 3(d) (LI bout).

Both groups experienced similar RPE for breathing and overall (MI: 7.0±3.0 vs. NMI: 6.0±2.0, p>0.05) throughout the exercise at both intensities (Fig 3(e) and 3(f)). However, significant differences were observed in RPE for legs during cycling at both intensities towards the end of the 30 min bout (MI: 8.0±2.0; NMI: 7.0±2.0 HI and MI: 7.0±3.0; NMI: 6.0±2.0 LI) [F(1,33.0) = 9.2, p<0.01] and for group x intensity [F(1,33.0) = 4.8, p<0.05]. Moreover, there was a notable difference for RPE in the legs [F(1,28.5) = 7.6, p = 0.01], breathing [F(1,28.9) = 9.2, p<0.01] and overall [F(1,28.7) = 11.8, p<0.01](Fig 3(e)) at P1 HI (MI: 6.0±3.0 legs, 5.0±3.0 breathing, 6.0±3.0 overall vs. NMI: 5.0±2.0 legs, 5.0±2.0 breathing, 5.0±2.0 overall) and indicated at P1 for LI (MI: 5.0±3.0 legs, 4.0±3.0 breathing, 4.0±3.0 overall vs. NMI: 4.0±2.0 legs, 5.0±2.0 breathing, 4.0±3.0 overall) (Fig 3(f)). Interestingly, the MI group appeared to experience a similar level of RPE for both intensities despite achieving lower workloads (W) to complete the exercise in comparison to the NMI group. The full set of data is available as supplementary material (S1 Table).

## Discussion

Children and adolescents with MI experienced higher perceived fatigue after both high and low intensity exercise despite having similar or lower physiological HR exercise responses during and following cycling. The MI group tolerated the short duration, HI exercise and the continuous lower-intensity endurance exercise similarly. Therefore, both approaches may serve as a feasible modality for improving fitness but the long-term benefits and sustainability of different exercise intensities still remains to be evaluated. We propose that perceived fatigue is limiting exercise for youth with MI and interventions to reduce perceived fatigue during exercise may positively impact on exercise engagement in this group.

Throughout the literature, it has been consistently shown that children with movement difficulties (i.e., pDCD and DCD) are disadvantaged to various degrees on exercise capacity and muscle strength. Individuals with MI perform less well on fitness parameters [15] of: aerobic power, muscle strength, endurance, anaerobic power and body composition [13, 19, 26]. Similar to previous findings examining submaximal and maximal exercise test measures [13, 26], it was observed that children and adolescents with MI exhibited a lower $\dot{\text{V}}{\text{O}}_{2\text{peak}}$ and PPO compared to their TD peers (MI: 157.0±61.0 vs. NMI: 216.0±57.0 W). As hypothesised, participants with MI had a reduced exercise capacity, demonstrating a significantly lower $\dot{\text{V}}{\text{O}}_{2\text{peak}}$ in comparison to NMI (MI: 31.5 vs. NMI: 36.0 ± 11.0 mL/kg·min). Furthermore, the $\dot{\text{V}}{\text{O}}_{2\text{peak}}$ for the MI group was below the cardiovascular fitness threshold [42] and as such, associated with an increased risk of obesity, type II diabetes and cardiovascular and metabolic conditions in adulthood [43].

Parallel to values reported by Faught et al. [26], HR_max_ or the term HR_peak_ used in this study was analogous between groups (MI: 170.0±25.0 and NMI: 180.0±17.0 bpm, p>0.05). However our findings deviated from the results in Morris et al. [13], who saw a significant mean HR difference of 12 bpm in children with higher MI (176 bpm) versus NMI (188 bpm). These differences may be due to the small sample size presented in the current study, which served as a limitation to the robustness of these findings. In addition, perceived competence to complete the task could also limit the results of the $\dot{\text{V}}{\text{O}}_{2\text{max}}$ test in children with MI. It is important to note that the MI group demonstrated a similar RPE to NMI despite significant differences in $\dot{\text{V}}{\text{O}}_{2\text{peak}}$ performance and exercise capacity. Thus, the limiting factors to exercise may be more perceptually related and central in origin [28]. Both groups exceeded an average RER of 1.06 at the end of the exercise test; however, RER was notably higher in NMI (1.34 in NMI vs. 1.20 in MI), which was different to the findings in Morris et al. (2013). Substrate utilisation and fat oxidation levels during the maximal exercise test were within the range previously reported in healthy children and adolescents [44]. Although the MI group demonstrated a significantly lower PPO there was no difference in the perception of effort (RPE) reported throughout and at test termination similar to Morris et al., [13]. All participants reported a RPE rating of 9 or 10 at the end of the exercise test.

Corroborating earlier studies [13, 26], participants with MI exhibited a reduced movement economy during the incremental bike test as illustrated by their $\dot{\text{V}}{\text{O}}_{\text{2}}$/PO relationship (MI: 13.3 ± 4.0 vs. NMI: 11.2 ± 2.0 mL/W) (Table 2). According to Wasserman et al. [45], $\dot{\text{V}}{\text{O}}_{\text{2}}$ kinetics normally rise at a rate of approximately 8.5–11 mL/min·W and are independent of sex, age, body mass or height in youth. Therefore, poorer coordination and inefficient movement patterns may contribute to the reduced exercise capacity in MI. Similar to the observations in this study, Faught et al., [26] recognised that children with pDCD were disadvantaged from the beginning of the incremental exercise protocol at low workloads (i.e., <40 W) and worked at a greater percentage of their $\dot{\text{V}}{\text{O}}_{\text{2}}$ compared to healthy controls throughout the test. As such, even at very low exercise intensities, children with poor motor proficiency/coordination may need to utilise more energy to carry out basic movements associated with maintaining proper posture and posture on the cycle ergometer [26]. This further suggests that youth with MI produce inefficient movements and may exercise at a higher metabolic rate to sustain the same level of workload relative to children without. Furthermore, we suspect the MI group would require more oxygen to exercise because they do not work their cardiovascular system sufficiently as represented by the similar O_2_ pulse (MI: 19.0 ± 0.03 vs. 20.0 ± 0.05 mL/beat) but significantly different PPO attained.

To our knowledge, this is the first study to investigate the impact of varying exercise intensities and the responses during the recovery phase, particularly following HI exercise, in adolescents with MI. As previously alluded in Morris et al. (2013), the evidence of children with MI to push themselves maximally warrants the potential applications of performing HI exercise on improving measures of health, muscle function and movement coordination. The results in this study revealed that the NMI group was able to tax the cardiovascular system sufficiently during the HI session as indicated by the 12% difference for HR_avg_ between the HI (157.0±14.0 bpm) and LI (143.0±17.0 bpm) cycling bouts. Conversely, the MI group did not appear to demonstrate considerable difference in HR measures between HI (140.0±18.0 bpm) and LI (133.0±18.0 bpm) cycling with only a 4% difference in percentage change from HR_max_ between intensities. These findings potentially suggest a smaller ventilatory threshold (VT), or point during exercise at which ventilation starts to increase at a faster rate than $\dot{\text{V}}{\text{O}}_{\text{2}}$, in adolescents with MI compared to NMI. For most individuals, this threshold lies at exercise intensities between 50% and 75% of $\dot{\text{V}}{\text{O}}_{2\text{max}}$ and is dependent on the person’s level of fitness [46]. Exercise intensity for both HI and LI workload was set relatively to each child and considering the high peak RER observed in the MI group and lower actual intensity performed, it is likely that children with MI are exercising at a lower intensity relative to their VT. A limitation of this study however, was that the exercise intensities for the HI and LI cycling bouts were not determined from the VT and instead, calculated as a percentage of PPO (PP_50%_ and PP_100%_). Although difficult to interpret in a minority of children, the VT is considered a useful method to determine aerobic fitness in children [46] and future studies should examine VT and $\dot{\text{V}}{\text{O}}_{2\text{max}}$ changes following different types of training [16].

In the last decade, findings have highlighted the relationship between lower motor competence and fitness performance [13, 14]; noting the influence of self-efficacy and perceived adequacy [17]. The findings in this study showed that the MI group did not experience any differences in perceived fatigue and leg muscle fatigue compared to NMI peers following either HI-or LI-exercise. These observations are similar to studies reporting that children experience less fatigue during short-burst activities and often request to repeat high-intensity exercises after their completion determined to improve their previous performance [31]. However, during higher intensity exercise, the MI group reported higher levels of perceived fatigue in legs and breathing even though they performed at a lower overall exercise intensity (but still the same relatively) and achieved high RER values at $\dot{\text{V}}{\text{O}}_{2\text{peak}}$. Therefore, as opposed to the findings observed in Morris et al. [13], the factors limiting exercise performance and perceived fatigue in MI may be more central in origin rather than metabolic or peripheral. This suggests that the lower-intensity aerobic exercise will be better tolerated in the MI group in comparison to HI. Noteworthy, all participants completed each session without any adverse events and interestingly, both MI and NMI groups anecdotally preferred the HI cycling bout to the LI bout. With this in mind, exposure to different exercise intensities may build up more self-confidence and self-efficacy to participate in PA [4].

Strengths of this study include objective measurement of physiological and perceptual variables during and following different exercise intensities, and the examination of recovery markers in MI and NMI adolescents. Ongoing growth and maturation can confound exercise interventions unless controlled adequately with well-matched controlled groups. In this acute study, only a small sample of participants (n = 38) were measured and therefore, caution must be taken when interpreting the robustness of the outcomes. There were no statistical differences found between MI and NMI groups on level of maturation or age and thus, no further analyses were undertaken to delineate potential sex differences at this time. Moreover, the wide age range of the participants and the unequal distribution of boys and girls pose limitations on interpreting the findings. Maturation and puberty may raise significant implications for the results and according to Tolfrey and Smallcombe (2017), may be an important factor on training effect in HIT studies. However, the authors acknowledge it is difficult to identify an independent sex effect that is not due to baseline differences in peak $\dot{\text{V}}{\text{O}}_{\text{2}}$ or maturation [25]. New research has drawn a correlation between significant sex differences in the underlying pathways connecting pDCD to internalising problems, indicating more mediating pathways through PA, BMI and global self-worth in girls, compared to boys [47]. Future research should expand on these findings and include larger sample sizes, taking into account sex, maturation and age in relation to physiological and perceptual exercise responses.

## Conclusion

The findings from this study highlight the physiological, perceptual and recovery responses to different exercise intensities in children and adolescents with and without MI. The exposure to both a HI intermittent bout and a continuous LI cycling bout demonstrated a lower exercise capacity in children with MI and a higher perception of physiological symptoms while performing at a lower intensity generally. Furthermore, the results from the incremental exercise test alongside measures of muscle strength and fatigue before and after exercise suggest that central factors could be the limiting factor to exercise tolerance in this group. Interestingly, differences for group and intensity were observed in the recovery period, yet the pattern of recovery was similar between MI and NMI, which may be crucial for devising suitable exercises and activity intensities. Although the number of participants in this sample was relatively small, the results from each acute session contribute to future interventions and exercise prescriptions targeting aerobic and anaerobic fitness, strength and power and general participation in physical activities. Overall, all participants successfully completed the high and moderately-low intensity cycling bouts however, more research investigating the implementation of HI and LI exercise during longer-term interventions is required to further elucidate the sustainability and tolerance of this type of activity. Whether short durations of high-intensity intermittent exercise can feasibly improve health and fitness levels and adherence to longer-term PA engagement in youth with MI still remains to be explored.

## Supporting information

S1 Table Supplementary table of data. (PDF)
