# Supplementary material for: The impact of high and low-intensity exercise in adolescents with movement impairment
Source: PLoS One. 2018 Apr 26;13(4):e0195944. doi: 10.1371/journal.pone.0195944 (PMC5919484; doi:10.1371/journal.pone.0195944)
Supplement: S1 Table — (PDF) [file pone.0195944.s001.pdf]

Supplementary Table 1.

ID	Age	Height (cm	Weight (kg	BMI	Tanner Scal	BOT-2 Shor	Standard S	%ile Rankir
P1	14	174.5	59.6	0.00196	5	55	34	6
P2	14	169.6	47.8	0.00166	3	67	39	14
P3	17	177	78.3	0.0025	5	65	37	10
P4	13	164	54.6	0.00203	5	59	35	7
P5	18	178.8	64.6	0.00202	5	70	40	16
P6	11	145.8	47.1	0.00222	5	47	31	3
P7	16	180	107.9	0.00333	5	57	34	6
P8	15	172.8	87.9	0.00294	5	61	36	8
P9	12	161.3	61.7	0.00237	4	60	37	10
P10	12	168	64.5	0.00229	3	60	35	7
P11	16	175	64	0.00209	5	66	38	12
P12	16	175.5	57.3	0.00186	5	61	36	8
P13	14	163.6	58.9	0.0022	4	68	38	12
P14	16	176.6	61.8	0.00198	5	57		
P15	12	165.7	45.6	0.00166	3	62	38	12
P16	16	173.4	59.5	0.00198	5	61	36	8
P17	14	163	54.9	0.00207	5	69	39	14
**NMI**								
P18	17	176.3	62.6	0.00201	5	73	48	42
P19	17	191.6	115.9	0.00316	5	65	43	10
P20	14	183.8	79	0.00234	5	72	43	24
P21	17	169.3	68.2	0.00238	5	77	47	41
P22	17	181.1	61.4	0.00187	5	78	48	42
P23	18	172.8	70	0.00234	5	73	42	21
P24	18	192	81.4	0.00221	5	77	46	35
P25	11	167	59.2	0.00212	2	65	41	18
P26	13	179.5	66.5	0.00206	3	72	45	31
P27	17	163.3	46.4	0.00174	5	79	51	54
P28	13	174.6	60.4	0.00198	5	77	52	58
P29	18	183.3	73.4	0.00218	5	76	45	31
P30	16	169.7	55	0.00191	5	81	56	73
P31	16	173.4	59.7	0.00199	5	80	53	62
P32	12	165.5	45.4	0.00166	3	70	44	27
P33	16	171.9	62	0.0021	5	70	41	18
P34	17	173.1	70.2	0.00234	5	82	57	76
P35	11	143	38.5	0.00188	3			
P36	14	183.2	84.7	0.00252	5	72	43	24
P37	18	177.1	75.3	0.0024	5			
P38	17	172.4	59.6	0.00201	5	80		

Supplementary Table 1.

Descriptive	VO2 Max (l	VO2 norma	VO2/Watt	VO2/HR	HRmax	Wattmax	RER	RER0W
MI	2.75468	46.2195	0.01059	0.01413	195	160	1.08	0.97
MI	1.43757	30.0747	0.01027	0.00917	165	140	1.25	1.08
MI	2.14114	27.3454	0.00824	0.01166	191	260	1.5	1.06
MI	1.9231	35.2216	0.01344	0.01292	159	140	1.09	1.07
MI	2.58356	39.9932	0.00994	0.01395	196	260	1.57	0.98
MI	1.14209	24.2482	15.2279	8.58715	133	75	1.02	0.79
MI	1.145	10.6117	0.01908	0.01205		60	1.02	1.03
MI	2.5793	29.3436	0.01075	0.01336	193	240	1.31	0.84
MI	1.56659	25.3904	0.01305	0.01059	148	120	1.04	0.98
MI	1.554	24.093	0.00971	0.00863	180	160	1.44	1.13
MI	1.8249	28.5141	0.01825	0.01508	121	100	1.04	1.35
MI	2.13026	37.1773	0.01065	0.01076	198	200	1.38	0.94
MI	1.425	24.129	0.01357	0.01056	135			
MI	2	39	0.01111	0.01093	183	105	1.12	1.03
MI	2.23	48.032	0.01394	0.01155	193	180	1	0.81
MI	2.107	35.4118	0.01505	0.01293	163	160	1.13	0.81
MI	1.722	31.3661	0.01076	0.01019	169	140	1.24	0.95
NMI	2.30788	36.8671	0.01648	0.01184	195	260	1.33	0.89
NMI	2.66105	22.9599	0.01109	0.01565	170	240	1.25	0.98
NMI	2.71673	34.3889	0.01235	0.01493	182	240	1.39	0.8
NMI	2.8481	41.761	0.01187	0.01595	178	240	1.31	0.89
NMI	2.71673	44.2464	0.01235	0.01493	187	240	1.4	0.8
NMI	1.44375	20.625	0.01031	0.00825	188	160	1.41	0.94
NMI	4.04691	49.7164	0.0119	0.02221	185	380	1.34	0.92
NMI	1.74912	29.546	0.01094	0.01003	178	160	1.24	0.93
NMI	3.1428	47.2602	0.01209	0.01599	197			
NMI	1.58446	34.1479	0.00903	0.00847	187	160	1.43	0.95
NMI	2.48046	41.0671	0.00954	0.01326	186	260	1.48	1.02
NMI	1.99325	27.156	0.01107	0.0127	157	180	1.4	1.01
NMI	3.01879	54.8871	0.01372	0.01502	201	220	1.37	0.98
NMI	3.02233	50.6253	0.01374	0.01504	220	220	1.37	0.98
NMI	1.56659	25.3904	13.0549	10.5851	148	200	1.28	0.98
NMI	1.65044	26.6201	0.01179	0.01022	162	140	1.34	1.01
NMI	3.56705	50.8126	0.01274	0.02015	177	280	1.41	1.01
NMI	0.769	19.974	12.8167	5.16107	149		1.09	0.93
NMI	2.069	24.4274	0.01293	0.01182	175	160	1.22	1.09
NMI	2.518	33.4396	0.01399	0.01455	173	180	1.25	1.11
NMI	2.374	39.8322	0.01187	0.01326	179	200	1.4	0.88

Supplementary Table 1.

RER20W	RPE1min-L	Breathing	Overall	RPE2min	B	O	RPE3min	B
0.8								
1.05	2			3			4	
1	1	1	1	2	2	2	2	2
0.92	1	1	1	3	4	5	5	6
0.95	1	1	1	1	1	1	1	1
0.78	1	1	1	1	3	1	3	3
1.17	2	4	4	6	8	7	10	8
1.03	1	2	1	1	2	2	1	2
0.88	1	2	3	3	4	4	4	4
1.19	1	1	1	2	3	2	2	3
1.26	1	2	1	3	5	2	5	6
0.98	1	1	1	1	1	1	1	1
	1	1	1	1	1	1	2	2
1.04	1	1	1	1	1	1	1	1
0.86	1	1	1	1	1	1	1	1
0.79	2	2	2	4	3	3	5	4
0.92	1	1	1	1	1	1	1	1
0.82	1			1			2	
0.94	1			1			1	
0.79	1			1			1	
0.81	2	1	1	2	1	1	4	3
0.78	1	1	1	2	2	2	2	2
0.9	1	1	1	1	1	1	1	1
0.96	1	1	1	1	1	1	1	1
0.91	1	2	2	3	3	3	3	4
	1	1	1	1	1	1	1	1
0.97	1	1	1	1	1	1	2	2
1	1	1	1	1	1	1	1	1
0.98	1	1	1	2	2	2	3	2
0.96	1	1	1	1	1	2	1	1
0.96	1	1	1	1	1	1	1	1
0.91	1	1	1	1	1	1	1	1
1.03	1	1	1	1	2	1	2	1
1.03	1	1	1	2	2	2	2	2
0.9	1	1	1	6	4	5	7	6
1.03	1	2	3	1	2	3	4	5
1.01	1	1	1	1	1	1	2	1
0.95	2	1	2	2	1	2	2	1

Supplementary Table 1.

O	RPE4min	B	O	RPE5min	B	O	RPE6min	B
	5			6			7	
2	3	3	3	4	4	4	5	5
5	5	7	6	7	8	7	7	8
1	1	2	2	1	2	2	2	2
3	6	6	6	9	10	10		
10	10	6	7	8	4	5		
2	2	3	3	3	3	3	3	4
4	5	5	5	6	7	7	7	7
2	3	3	3	4	3	4	5	3
4	6	6	5	7	8	6	7	7
1	1	1	1	1	2	1	1	2
2	2	3	3	4	4	3	6	6
1	1	1	1	1	1	1	1	1
1	1	1	1	6	1	1	7	3
4	8	6	6	8	6	6	9	6
1	2	2	2	2	2	2	3	3
	2			3			3	
	1			1			1	
	1			2			2.5	
3	5	6	6	6	5	4	7	6
2	2	2	2	3	3	3	4	4
1	2	1	2	3	3	3	5	5
1	1	1	1	1	1	1	1	2
4	5	4	4	5	5	5	5	6
1	1	1	1	1	1	1	3	2
2	2	2	2	2	2	2	3	3
1	1	1	1	1	1	1	2	2
2	3	2	3	4	3	3	5	4
1	1	1	1	1	1	1	2	2
1	2	2	2	2	3	3	3	3
1	1	1	1	2	2	2	2	2
2	2	1	2	3	2	3	6	4
2	2	2	2	3	3	3	3	3
6	8	8	8	9	10	10	10	8
6	7	8	10	7	8	9	5	7
1	2	1	1	2	2	1	3	2
2	2	1	2	2	2	2	3	2

Supplementary Table 1.

O	RPE7min	B	O	RPE8min	B	O	RPE9min	B
	8			9				
5	6	5	6	6	5	6	7	6
9	9	10	9	1	10	9	10	10
2	2	3	3	3	3	3	4	3
3	4	5	4	5	5	5	7	7
8	7	7	8					
4	6	4	5	6	5	6	8	8
7	8	8	8	7	7	7	6	6
1	1	2	1	2	2	1	2	2
6	7	8	8	10	10	10		
1	1	1	1	1	1	1	1	1
1	8	5	2	9	6	3	10	8
6	9	6	6	10	7	6		
3	7	5	6	7	5	6	9	9
	4			5			6	
	2			2			3	
	3			4			5	
4	3	5	4	7	6	5	7	7
4	5	5	5	5	5	5	6	6
5	8	8	8	9	8	9		
2	1	2	2	2	2	2	3	3
5	7	6	7	8	9	9	5	6
1	4	1	2	4	2	3	4	2
3	4	4	4	6	6	6	8	8
2	2	2	2	2	2	2	3	3
4	6	4	5	7	5	6	8	6
2	2	2	2	3	3	3	4	3
3	4	4	4	5	5	5	6	6
2	3	3	3	3	4	4	5	4
6	9	6	8	10	7	10		
3	4	4	4	4	4	4	5	4
9	10	7	8					
4	4	9	6	3	5	2	4	7
2	5	3	3	7	4	3	7	5
3	3	2	3	4	3	4	5	5

Supplementary Table 1.

O RPE10min B O RPE11min B O RPE12min B

7	8	7	8	8	7	8	9	8
10								
4	5	4	5	5	4	5	6	5
7	7	8	7	9	9	9	9	9
8								
6								
2	3	2	2	3	3	3		
1	1	1	1					
5								
9								
	7			8			8	
	4			5			6.5	
	6			6.5			7	
7	8	8	8	7	8	8	10	10
6	7	7	7	8	8	7	9	8
3	3	3	3	3	4	3	3	4
7								
3	4	2	3	4	2	3	6	3
8	9	9	9					
3	4	3	3	4	3	3	5	4
9	9	6	9					
4	5	5	4	6	7	6	7	7
6	8	8	9	9	9	9		
5	7	7	7	9	9	9	10	10
4	5	5	5	6	5	5	6	6
3								
4	10	6	7					
5	7	6	7	8	9	9		

Supplementary Table 1.

**O**	**RPE13min**	**B**	**O**	**RPE14min**	**B**	**O**	**RPE15min**	**B**
9	10	9	10					
6	8	6	7	9	7	8		
9	10	10	10					
	9							
	8			9			9	
10								
8.5	9	9	9					
4	4	4	4	5	5	5	5	6
4	7	5	7	9	8	9		
4	7	4	5	7	4	5		
7	8	9	8					
10								
6	7	7	7	7	8	8	9	9

Supplementary Table 1.

O **RPE16min B** **O** **RPE17Legs B** **O** **RPE18Legs B**

9

6 6 7 7 7 8 8 8 8

9

Supplementary Table 1.

**O**		**RPE19Legs B**	**O**		**RPE20Legs B**	**O**			**Post1min**	**B**	
									1.5		
									5		5
									8		4
									10		10
									7		5
									5		7
									2		1
									7		7
									4		1
									1		1
									5		5
									9		6
									4		4
									10		10
									6		5
									8		8
	8	9	9	9	10	10	10		4		6
									4		7
									6		2
									4		6
									6		3
									7		4
									3		2
									1		1
								\		\	
									10		7
									1		7
									6		3
									8		5

Supplementary Table 1.

O	Post3min	B	O	HR1min	HR2min	HR3min	HR4min	HR5min
				93	106	112	109	128
5	3	1	3	85	94	93	106	117
				84	94	103	113	123
6	6	2	4	92	98	98	107	107
10	3	10	10	92	82	94	107	117
				81	92	93	86	
5				103	105	108	116	129
8	7	6	6	82	113	113	123	127
2	1	2	1	100	140	135	144	151
7	6	6	6	91	113	103	111	112
				71	110	106	113	119
1	2	1	1	96	105	105	116	120
1	1	1	1	140	139	137	162	192
1	2	2	1	115	121	147	161	158
5	8	6	6	101	114	120	132	145
4	2	1	1	112	119	127	134	149
				91	111	117	119	
				97	101	102	106	112
				109	109	109	115	127
10	6	9	1	88	89	96	127	124
5	5	4	4	75	74	85	88	95
8	3	2	2	126	133	146	155	164
5	2	2	2	85	106	102	101	104
6	2	4	3	89	101	108	119	126
3	1	1	1	98	123	122	124	129
6	6	3	4					
							117	125
5	5	2	3	109	117	116	117	122
5				118	125	129	128	
0				106	109	121	127	140
				80	98	103	107	117
1	1	1	1	90	120	131	131	131
\	\	\	\	92	98	108	110	125
8				92	125	127	129	150
5	2	5	9	109	115	118	123	124
2	3	2	1	100	106	108	117	118
6	8	5	6	111	129	123	124	130

Supplementary Table 1.

HR6min	HR7min	HR8min	HR9min	HR10min	HR11min	HR12min	HR13min	HR14min
135	140	158						
123	132	139	150	158	172	179	183	191
132	142	159						
125	125	136	145	148	160	170	180	190
130								
135	145	154	161	173	181	189		
147	152							
155	176	179	180					
122	110							
132	142	163	177	183	197			
121	130	134						
200			200	201				
172	185	189	192					
156	165							
155				169				
139	149	161	172	178	187	191		
118	121	130	140	142	153	167	171	
144	146	151	157	164	168	173	178	182
131	139	144	147	161	171	178		
107	127	136	154	163	172	180	183	187
170	178	188						
116	118	127	131	139	144	150	156	155
139	151	168	178					
137	147	151	167	168	179	186	192	197
127	135	147	154	159	175	182	186	
130	137	139	147	155				
135	142	161	171	188	197	205	206	
150	159	176	185	193	201	173	161	140
131	142	151	170	182	188	194		
146	152	162						
128	130	144	146	157	166	175	177	183
152								
141	152	162	172					
130	137	144	161	167				
138	155	164	164	178	179			

Supplementary Table 1.

**HR15min**	**HR16min**	**HR17min**	**HR18min**	**HR19min**	**HR20min**	**Post1min**	**Post3min**	**HI**
								17/12/13
						114		18/02/20:
						128	103	41762
								41765
196						145	122	15/03/14
						77	60	15/04/14
						\	\	17/07/20:
						154		41856
						120	94	13/06/14
						135	105	19/07/20:
						91	80	15/05/14
								41954
						88	87	41894
							115	27/01/20:
						170	142	16/12/20:
						138	111	42066
						139	121	14/04/20:
								17/12/13
								41731
187								41793
						136	93	25/02/14
						134	97	27/02/14
						178	125	41793
155	169	168	180	182	185	122	86	23/03/14
						113	84	27/03/14
						160	125	25/03/14
								41794
						116	110	24/04/14
						135	120	17/04/14
						176	159	16/04/14
						88		27/04/14
						141	128	41188
						142	112	16/09/20:
190								
						105		21/10/20:
						131	109	24/02/20:
						141	127	16/04/20:
						166	145	

Supplementary Table 1.

WATTS	RPE0	B	O	RPE5	B	O	RPE10	B
160	1			5			5	
140	1	1	1	4	4	4	5	5
280	1	1	1	3	2	3	5	3
140	1	1	1	3	3	4	6	5
260	1	1	1	1	3	2	3	4
75	1	1	1	10	10	10	10	10
60	3	1	2	4	1	3	5	2
11	1	1	1	3	3	3	4	4
120	1	1	1	3	2	2	3	2
160	1	1	1	3	2	2	5	3
160	1	1	1	3	3	3	4	4
180	1	1	1	1	2	1	1	2
105	1	1	1	4	4	4	7	7
180	1	1	1	1	1	1	1	1
160	1	1	1	3	1	1	5	2
120	1	1	1	5	3	3	7	5
160	1	1	1	5	4	4	5	4
260	3			3			4	
240	1			3			7	
300	1	1	1	5	3	4	5	4
240	1	2	1	10	6	10	10	10
240	1	1	1	4	3	4	6	5
200	1	1	1	4	4	4	5	5
380	1	1	1	1	2	1	2	3
160	1	1	1	4	5	5	6	7
260	1	1	1	4	2	3	5	2
160	1	1	1	5	4	4	6	6
260	1	1	1	2	1	2	3	2
180	2	2	2	3	3	3	5	5
240	1	1	1	3	3	3	3	3
220	1	1	1	3	3	3	4	5
220	1	1	1	4	4	4	5	5
140	1	1	1	3	2	3	4	2
	1	1	1	3	2	2	4	3
60	2	2	2	6	5	7	8	9
160	3	4	7	8	5	7	7	8
180	1	1	1	3	2	1	6	3

Supplementary Table 1.

O	RPE15	B	O	RPE20	B	O	RPE25	B
	5			6			6	
5	5	5	5	6	5	6	6.5	6
5	7	5	7	8	6	8	9	6
7	7	6	8	8	7	9	9	8
4	5	5	5	6	5	6	7	7
10	10	10	10	10	10	10	10	10
3	5	2	4	6	3	4	6	3
4	5	5	5	5	5	5	6	6
4	3	2	4	8	9	9	10	10
4	6	4	5	7	4	5	8	5
4	5	5	5	7	7	7	10	9
1	2	2	2	2	2	3	2	2
7	8	8	8	9	7	8	9	8
1	2	2	2	2	2	1	1	2
2	6	3	2	7	1	1	7	4
5	8	6	6	9	7	7	9	8
5	6	4	5	8	6	7		
	4.5			6	6	5	4	
	9							
5	7	4	5	8	5	7	9	5
10	10	9	10	10	10	10	10	9
5	6	6	6	7	6	6	7	7
5	7	5	6	8	6	8	8	7
2	3	3	3	3	5	4	5	6
6	7	9	8	8	7	9	9	8
3	5	3	4	6	3	4	5	3
6	6	5	6	7	6	7	7	6
3	3	3	3	5	4	4	6	5
4	6	5	4	7	5	5	7	6
3	4	5	5	5	5	5	6	7
5	5	6	6	6	7	7	7	8
5	7	7	7	6	7	6	8	8
3	4	4	4	5	3	4	6	4
3	5	4	4	6	5	5	6	6
8	9	9	9					
5	8	5	9	6	4	7	7	8
4	4	4	3	5	3	4	5	3

Supplementary Table 1.

O	RPE30	B	O	RPEPost1	B	O	RPEPost3	B
	6			1				
6	7	6	7	7	7	7	6	5
9	9	7	9	8	4	8	7	2
9	8	7	8	5	3	3	2	1
7	7	6	7	6	4	5	5	2
10	10	10	10	10	10	10	10	1
4	7	2	4	6	1	3	5	2
6	6	6	6	6	5	5	4	3
10	10	10	10	7	10	10	3	3
6	8	5	6	5	6	6	1	3
10	10	10	10	8	8	8	7	6
3	3	3	3	3	3	3	3	3
9	9	8	8	8	5	7	6	4
2	2	2	2	1	1	1	1	1
3	8	5	2	9	1	1	8	1
8	10	8	8	10	8	8	8	6
				4	1	3	1	1
	5	4	5	1				
	9	9	9	1				
7	8	4	6	6	3	4	3	1
10	10	10	10	9	9	10	10	9
7	8	8	8	6	5	5	4	2
8	7	6	7	7	5	5	3	2
5	6	7	6	6	6	6	6	6
10	9	8	9				2	1
4	7	4	5	6	3	4	5	1
7	7	5	6	7	4	6	6	2
5	6	5	5	3	2	2	3	1
6	6	6	5	5	5	4	4	3
7	7	7	7	6	6	6	2	2
8	8	9	9	5	7	6	5	5
8	9	9	9	9	9	9	2	1
5	7	5	6	2	1	1	1	1
6	7	6	6	5	5	5	5	3
	9	9	9	9	10	10	10	8
4	8	4	9	5	8	7	6	5
4	3	4	3	3	2	3	3	1

Supplementary Table 1.

O	RPEpost7	B	O	HR0	HR5	HR10	HR15	HR20
				112	162	163	163	180
5				73	140	155	152	139
7	7	1	7	79	156	166	174	176
2	1	1	1	83	123	143	125	140
4	4	1	2	59	155	161	170	176
10	1	1	1	75	110	102	106	118
3	3	2	3	86	107	106	104	110
3	2	1	1	85	146	142	153	149
3	4	3	3	110	138	151	153	151
3	1	1	1	92	162	162	159	158
6	5	5	5	96	115	118	131	136
3	2	2	2	105	157	146	152	163
5	5	3	4	93	123	133	136	136
1	1	1	1	63	157	143	151	152
1	1	1	1	109	145	168	179	175
6	6	3	3	71	140	146	136	140
1	1	1	1	100	162	188	191	147
				106	167	166	168	168
				103	162	160	139	
2	2	1	1	97	159	162	164	170
10				76	156	158	147	161
3				71	159	179	171	176
2	2	1	2	94	180	182	180	181
6	4	4	4	91	161	167	164	168
2	1	1	1	91	160	161	161	162
3	2	1	1	79	162	171	166	171
4	4	1	2	87	182	184	187	185
2	2	1	1	102	161	157	172	173
3	2	2	2	98	126	123	135	137
2	1	1	1	102	174	191	194	189
5	3	4	3	112	194	200	188	197
2	1	1	1	93	171	178	175	185
1	1	1	1	91	137	163	154	161
4	5	3	3	76	145	158	159	160
9	9	7	8	108	156	148	149	
7	7	4	9	105	145	163	155	158
2	2	1	2	98	144	148	150	155
				142	170	178	178	181

Supplementary Table 1.

HR25	HR30	HR AVG	%HR Chang	HRPost1mi	HRPost3mi	HRPost7	%HRP1	%HRP3
179	183	163.143	83.663				0	0
144	155	136.857	82.9437	135	115		184.932	157.534
181	185	159.571	83.5453	165	118	106	208.861	149.367
137	146	128.143	80.593	101	90	81	121.687	108.434
179	180	154.286	78.7172	165	122	118	279.661	206.78
117	126	107.714	80.9882	117	93	75	156	124
103	108	103.429		86	69	79	100	80.2326
158	165	142.571	73.8712	151	112	105	177.647	131.765
144	149	142.286	96.139	118	110	86	107.273	100
158	176	152.429	84.6825	122	103	98	132.609	111.957
124	133	121.857	100.708	111	101	96	115.625	105.208
166	160	149.857	76.0696	147	103	98	140	98.0952
139	132	127.429	94.3915	129	102	127	138.71	109.677
139	148	136.143		114	108	119	180.952	171.429
189	182	163.857	84.9001	148	140	134	135.78	128.44
132	130	127.857		93	92	91	130.986	129.577
		157.6		144	130	110	144	130
169	170	159.143	81.6117	138			130.189	0
		141	82.9412	130			126.214	0
164	170	155.143	85.2433	?	?	?		
159	160	145.286	81.6212	124	93		163.158	122.368
177	185	159.714	85.4087	154	128		216.901	180.282
185	189	170.143	90.5015	159	130	115	169.149	138.298
180	175	158	85.4054	147	139	106	161.538	152.747
162	166	151.857	85.313		81	74	0	89.011
173	183	157.857	80.1305	159	138	116	201.266	174.684
193	205	174.714	93.4301	197	132	119	226.437	151.724
177	176	159.714	85.8679	137	106	101	134.314	103.922
143	143	129.286	82.3476	133	116	101	135.714	118.367
198	198	178	88.5572	184	134	129	180.392	131.373
185	199	182.143	82.7922	163	116	121	145.536	103.571
180	182	166.286		130	101	93	139.785	108.602
160	171	148.143	91.4462	144	120	120	158.242	131.868
158	160	145.143	82.0016	150	85	79	197.368	111.842
		140.25	94.1275	121	108	100	112.037	100
160	160	149.429		114	112	109	108.571	106.667
151	155	143		118	105	106	120.408	107.143
182	185	173.714					0	0

Supplementary Table 1.

%HRP7	%HRAvg	BP	HR	LI Watts	RPE0	B	O	RPE5
0	145.663	119/61	78		1			3
0	187.476	110/69	85	70	1	1	1	3
134.177	201.989	131/72	80	140	1	1	1	2
97.5904	154.389	118/81	70	70	1	1	1	5
200	261.501	112/61	53	130	1	1	1	3
100	143.619	90/39	56	38	1	1	1	7
91.8605	120.266	145/64	72	30	3	3	3	4
123.529	167.731	133/75	65	110	1	1	1	2
78.1818	129.351	115/80	84	60	1	1	1	3
106.522	165.683	132/73	94	80	1	1	1	2
100	126.935	135/68	80	80	1	1	1	2
93.3333	142.721	119/71	68	90	1	1	1	1
136.559	137.02	121/62	85	53	1	1	1	4
188.889	216.1	118/55	85	90	2	2	2	2
122.936	150.328	127/76	109	80	1	1	1	1
128.169	180.08	125/58	97					
110	157.6	110/60	92	80	1	1	1	4
0	150.135	124/73	66		2			2
0	136.893	143/79	80		1			2
	159.941			150	1			5
0	191.165	128/79	71	120	1	1	1	5
0	224.95	120/68	110	120	1	1	1	3
122.34	181.003	115/72	72	100	1	1	1	5
116.484	173.626	128/72	79	190	1	1	1	1
81.3187	166.876	116/66	77	80	1	1	1	4
146.835	199.819	125/60	60	130	1	1	1	2
136.782	200.821	103/71	89	80	1	1	1	3
99.0196	156.583	99/58	88	130	1	1	1	1
103.061	131.924	115/68	89	90	1	1	1	3
126.471	174.51	123/60	76	120	1	1	1	1
108.036	162.628	133/59	79	110	1	1	1	2
100	178.802	102/50	72	110	1	1	1	3
131.868	162.794	134/70	88	70				
103.947	190.977	113/74	64	140	2	2	2	4
92.5926	129.861	99/55	99	30	1	1	1	5
103.81	142.313	133/77	90	80	1	5	7	4
108.163	145.918	116/68	88	90	1	1	1	2
0	122.334							

Supplementary Table 1.

	RPE10	B	O	RPE15	B	O	RPE20	
		5			5			6
2	3	5	4	5	7	5	6	8
1	2	4	3	4	6	5	6	7
5	7	8	9	9	9	10	10	10
1	2	4	1	3	4	1	3	5
7	7	10	5	5	10	10	10	10
3	4	6	4	7	7	5	7	8
3	3	4	4	4	5	5	5	6
2	3	3	5	5	6	7	9	6
1	2	1	3	3	4	4	4	6
3	3	3	4	5	5	6	7	8
1	1	1	2	1	1	1	1	2
3	4	7	5	6	9	9	9	9
2	2	2	1	2	3	2	2	2
2	1	3	2	1	4	3	2	5
4	4	6	4	5	5	3	4	4
		2			5			4
		3			4			5
		6			7			6
7	10	8	8	10	9	10	10	10
2	2	3	3	3	4	3	3	5
4	5	8	6	8	9	7	9	10
2	2	1	3	2	2	4	3	4
3	3	5	5	5	6	7	8	10
1	1	3	2	1	3	2	1	3
3	3	5	4	4	6	5	5	6
1	1	3	1	3	4	2	3	3
2	3	6	3	6	5	3	4	6
1	1	1	2	2	3	2	3	3
1	1	3	2	3	3	3	3	4
3	3	4	4	4	4	5	4	5
3	3	4	4	4	4	5	5	6
4	6	6	7	8	8	9	10	9
6	3	2	5	7	7	6	4	6
1	1	2	1	2	3	1	2	4

Supplementary Table 1.

B	O	RPE25	B	O	RPE30	B	O	RPEPost1
		6			4	5	4	
6	7	8	7	8	9	7	8	9
5	7	7	5	7	7	5	7	6
10	10	10	10	10	10	10	10	7
2	4	6	2	5	5	1	4	4
10	10	10	10	10	10	10	10	1
5	9	9	5	10	10	5	9	6
6	6	7	4	6	7	5	6	6
7	8	8	9	9	9	9	9	10
5	5	7	6	7	8	7	8	4
8	9	10	10	10	10	10	10	9
1	2	2	2	2	2	2	2	1
9	9	9	9	9	9	9	9	7
2	2	2	2	2	2	2	3	1
4	1	7	5	3	8	5	1	5
4	4	4	4	4	4	4	4	2
		5			5	5	5	
		5			6	5	5	
		5.5			5	6	5	
10	9	9	9	9	10	10	10	10
3	4	5	3	4	6	4	5	5
7	9	9	7	9	9	7	9	8
5	4	4	5	5	4	5	4	3
10	10				10	10	10	
2	1	4	1	1	4	1	1	3
5	5	6	5	5	6	5	5	6
3	3	4	3	3	4	3	3	3
3	5	4	3	4	3	2	3	2
3	3	4	3	3	3	3	3	1
3	3	4	3	4	4	3	3	3
6	6	7	7	7	8	8	8	7
5	5	6	6	6	7	6	6	5
9	10	9	9	10	9	9	10	5
8	4	4	7	5	6	4	9	5
2	3	4	2	4	4	2	3	3

Supplementary Table 1.

B	O	RPEPost3	B	O	RPEpost7	B	O	HR0
		1						105
8	8	6	5	5	5	3	5	93
3	6	6	1	4				73
7	5	7	6	5	1	1	1	79
1	3	4	1	3	1	1	1	106
1	1	1	1	1	1	1	1	64
4	5	6	4	5	3	3	3	106
5	5	4	1	2	2	1	1	94
8	8	10	9	7	6	2	6	91
3	4	1	2	1	1	1	1	155
9	9	10	10	10	9	9	9	101
1	1	1	1	1	1	1	1	89
8	8	6	1	4	6	1	3	99
1	1	1	1	1	1	1	1	105
1	1	4	1	1	7	1	1	109
1	1	1	1	1	1	1	1	101
		1						139
		1						117
								162
10	10	10	10	10	10	10	10	75
2	3	4	1	2				87
5	7	6	3	4				93
4	3	4	3	3	4	2	2	88
								82
1	1	3	1	1	2	1	1	95
5	5	6	3	4	4	2	3	97
3	2	2	1	1	1	1	1	64
1	2	2	1	1	1	1	1	85
2	2	1	1	1	1	1	1	78
2	2	3	2	2	3	1	1	91
4	5	4	1	2	1	1	1	98
4	4	4	3	3	3	2	2	109
6	8	4	4	4	7	7	7	121
3	6	6	7	5	7	1	8	88
1	2	2	1	3	2	1	1	97

Supplementary Table 1.

HR5	HR10	HR15	HR20	HR25	HR30	HR AVG	%HR Chang	HRPost1
151	157	163	163	160	159	151.143	77.5092	142
125	135	140	143	140	141	131	79.3939	119
143	156	164	164	169	173	148.857	77.9357	139
120	122	123	118	118	125	115	72.327	92
153	160	164	167	166	169	155	79.0816	152
125	124	135	130	137	131	120.857	90.87	113
97	92	95	100	102	98	98.5714		89
134	132	132	136	134	139	128.714	66.6913	130
136	128	122	136	131	134	125.429	84.749	118
153	153	152	160	156	174	157.571	87.5397	127
126	122	120	123	127	125	120.571	99.6458	104
131	135	133	139	143	139	129.857		109
128	124	121	117	120	119	118.286		99
128	130	130	134	125	131	126.143		93
162	164	167	169	169	170	158.571		164
167	177	174	75	174	190	151.143		145
159	159	160	160	170	170	159.571	81.8315	124
123	120	132	112	132	133	124.143	73.0252	108
196	151	152	158	161	165	163.571	89.8744	141
142	148	145	152	147	157	138	77.5281	126
126	142	146	151	151	155	136.857	73.1856	137
174	180	184	185	188	189	170.429	90.6535	169
137	147	149	147	148	151	138.143	74.6718	124
144	148	156	147	160		139.5	78.3708	107
148	162	163	165	168	169	152.857	77.5925	151
172	181	189	189	193	194	173.571	92.8189	180
135	141	147	147	142	149	132.143	71.0445	136
118	125	127	123	123	121	117.429	74.7953	104
158	165	167	176	183	182	158.429	78.8202	152
146	154	153	167	161	167	148.429	67.4675	158
71	148	158	172	170	172	141.286		122
126	132	138	139	141	144	132.714	74.9798	91
124	128	128	132			126.6	84.9664	118
135	135	141	137	142	143	131.571		110
126	132	138	139	144	141	131		126

Supplementary Table 1.

HRPost3	HRPost7	HRAVGPos	%HRPost1	%HRPost3	%HRPost7	%HRAvg	BP	HR
		142.865	135.238	0	0	143.946	109/69	89
103	96		127.957	82.4	103.226	140.86	103/57	83
114			190.411	79.7203	0	203.914	139/90	82
78	70		116.456	65	88.6076	145.57	131/61	70
126	103		143.396	82.3529	97.1698	146.226	112/69	80
94	77	111.811	176.563	75.2	120.313	188.839	106/55	64
70	70		83.9623	72.1649	66.0377	92.9919	145/64	72
110	96	119.367	138.298	82.0896	102.128	136.93	134/75	71
101	99	117.181	129.67	74.2647	108.791	137.834	128/61	66
103	107	112.333	81.9355	67.3203	69.0323	101.659	120/64	94
105	101	103.333	102.97	83.3333	100	119.378	134/83	99
98	93	100	122.472	74.8092	104.494	145.907	111/61	63
98	99		100	76.5625	100	119.481		
97	99		88.5714	75.7813	94.2857	120.136	113/63	77
148	124		150.459	91.358	113.761	145.478	116/71	91
123	113		143.564	73.6527	111.881	149.646	105/91	101
		148.24	89.2086	0	0	114.8	111/62	79
		117.417	92.3077	0	0	106.105	137/62	85
			87.037	0	0	100.97	137/62	62
88	93		168	117.333	124	184	147/84	70
114			157.471	131.034	0	157.307		72
129			181.72	138.71	0	183.257	102/64	83
120	85		140.909	136.364	96.5909	156.981	125/58	67
82	79		130.488	100	96.3415	170.122		
118	110	139.954	158.947	124.211	115.789	160.902	118/61	54
155	131		185.567	159.794	135.052	178.94	103/74	102
111	89	122.016	212.5	173.438	139.063	206.473		74
94	90	108.519	122.353	110.588	105.882	138.151	128/71	91
126	121	145.437	194.872	161.538	155.128	203.114	136/66	65
123	103	136.575	173.626	135.165	113.187	163.108	122/56	62
121	95	133.481	124.49	123.469	96.9388	144.169	109/51	66
73	68	77.3333	83.4862	66.9725	62.3853	121.756	118/44	61
106	102	108.667	97.5207	87.6033	84.2975	104.628	98/61	96
103	93		125	117.045	105.682	149.513	132/85	83
105	98	109.667	129.897	108.247	101.031	135.052	115/72	86
